# Repercussions of the COVID-19 pandemic on breast cancer actions in a Brazilian state

**DOI:** 10.17533/udea.iee.v42n2e04

**Published:** 2024-07-03

**Authors:** Paula Danniele dos Santos Dias, Mary Elizabeth de Santana, Vera Lúcia de Azevedo Lima

**Affiliations:** 1 Nurse, Master. Regional Hospital Abelardo Santos. Email: pauladanniele14@gmail.com. Corresponding author Universidade Federal do Pará Regional Hospital Abelardo Santos Brazil pauladanniele14@gmail.com; 2 Nurse, Ph.D. Associate Professor I. Email: marybete@ufpa.br Universidade Federal do Pará Brazil marybete@ufpa.br; 3 Nurse, Ph.D. Associate Professor. Email: veraluci@ufpa.br Universidade Federal do Pará Brazil veraluci@ufpa.br; 4 Federal University of Pará Belém - Pará - Brazil Universidade Federal do Pará Federal University of Pará Belém Pará Brazil

**Keywords:** COVID-19, SARS-CoV-2, mammogram, breast neoplasms, breast carcinoma in situ., COVID-19, SARS-CoV-2, Mamografía, Neoplasias de la Mama, Carcinoma de Mama in situ, COVID-19, SARS-CoV-2, mamografia, neoplasias da mama, carcinoma da mama in situ.

## Abstract

**Objective.:**

To analyze whether the COVID-19 pandemic had an impact on the screening, diagnosis and treatment of breast cancer in women up to 50 years of age in the state of Pará.

**Methods.:**

Retrospective, cross-sectional study with a quantitative approach, using data from the Information Technology Department of the Brazilian Unified Health System. (DATASUS). The number of exams carried out in the pre-pandemic (2018-2019) and pandemic (2020-2021) period was analyzed based on the percentage variation, application of the chi-square test and G test for the time of exams and start time of treatment.

**Results.:**

During the pandemic period, there was a greater number of screening mammograms (+3.68%), cytological (+23.68%), histological (+10.7%) and a lower number of diagnostic mammograms (-38.7%). The time interval for carrying out the exams was up to 30 days for screening and diagnostic exams and more than 60 days to start treatment during the pandemic period.

**Conclusion.:**

Although the results indicate an increase in the number of screening and diagnostic procedures for breast cancer during the pandemic period, with the exception of diagnostic mammography, when considering probability values, the study points out that statistically the COVID-19 pandemic did not interfere with actions of breast cancer, in women over 50 years of age, in the state of Pará. Considering the autonomy of nursing and its role in public health, it is up to the professionals who are in charge of primary care programs to implement contingency plans in periods of crisis so that the population is not left unassisted.

## Resumen

**Objetivo.:**

Analizar si la pandemia de COVID-19 tuvo impacto en el tamizaje, diagnóstico y tratamiento del cáncer de mama en mujeres de 50 años y más del Estado do Pará-Brasil.

**Métodos.:**

Estudio retrospectivo, transversal, con abordaje cuantitativo, en el que se utilizaron los datos del Departamento de Informática del Sistema Único de Salud de Brasil (DATASUS). Se comparó el número de exámenes realizados y el tiempo para el inicio de tratamiento en los períodos prepandémico (2018-2019) y pandémico (2020-2021).

**Resultados.:**

Se observó un mayor número de mamografías de cribado (+3.68%), citologías (+23.68%) e histologías (+10.7%) y un menor número de mamografías diagnósticas (-38.7%) en el período pandémico. El tiempo para la realización de las pruebas fue de hasta 30 días para el cribado y diagnóstico y de más de 60 días para el inicio del tratamiento durante el período pandémico.

**Conclusión.:**

Aunque los resultados indican un aumento del número de procedimientos de cribado y diagnóstico del cáncer de mama en el periodo pandémico, con la excepción de la mamografía diagnóstica, cuando consideramos los valores de p) el estudio muestra que la pandemia COVID-19 estadísticamente no interfirió en las acciones preventivas contra el cáncer de mama en mujeres de 50 años y más en el estado de Pará. Teniendo en cuenta la autonomía de la enfermería y su papel en la salud pública, corresponde a los profesionales responsables de los programas de atención primaria implementar planes de contingencia en tiempos de crisis para no dejar desatendida a la población.

## Resumo

**Objetivo.:**

Analisar se a pandemia da COVID-19 repercutiu no rastreamento, diagnóstico e tratamento do câncer de mama em mulheres paraenses a partir de 50 anos.

**Métodos.:**

Estudo retrospectivo, transversal, de abordagem quantitativa, com utilização de dados do Departamento de Informática do Sistema Único de Saúde brasileiro. (DATASUS). Analisou-se o número de exames realizados no período pré-pandemia (2018-2019) e pandêmico (2020-2021) com base na variação percentual, aplicação do teste qui quadrado e teste G para o tempo de realização de exames e tempo de início de tratamento.

**Resultados.:**

Observou-se no período pandêmico maior quantitativo de mamografias de rastreamento (+3.68%), citológicos (+23.68%), histológicos (+10.7%) e menor registro de mamografias diagnósticas (-38.7%). O intervalo de tempo para realização dos exames foi de até 30 dias para os exames de rastreamento e diagnóstico e tempo maior que 60 dias para início de tratamento no período pandêmico.

**Conclusão.:**

Embora os resultados indiquem aumento no quantitativo de procedimentos de rastreamento e diagnósticos para o câncer de mama no período pandêmico, com exceção da mamografia diagnóstica, ao considerarmos os valores de probabilidade, o estudo aponta que estatisticamente a pandemia da COVID-19 não interferiu nas ações do câncer de mama, em mulheres a partir de 50 anos, no Estado do Pará. Considerando a autonomia da enfermagem e sua atuação na saúde pública, cabe aos profissionais que estão à frente dos programas da atenção básica implementar planos de contingência em períodos de crise para que a população não fique desassistida.

## Introduction

Cancer is the leading cause of death before the age of 70 in several countries, with its incidence and mortality increasing on the world scenario.^1^Breast cancer is the most common among women, the second most frequent and the fifth leading cause of cancer death in the world. The global estimate for the year 2020 was 2.3 million new cases of breast carcinoma, representing 11.7% of all cancer cases in the world. Breast cancer ranks first in incidence in most countries and in mortality in 110 countries, accounting for 1 in 4 new cases and 1 in 6 cancer deaths.^1^Its incidence is higher both in countries with a high Human Development Index (HDI) and in countries with a low or medium HDI.^1^

In Brazil, in 2019, the estimated rate of breast cancer was 56.33 per 100,000 women.^2^In the northern region of the country, the estimated number of new cases of breast cancer for the year 2020/2022 was 1,970 cases, of these, 780 cases in the state of Pará.^3^For the 2023/2025 triennium, it is estimated that there will be 73,610 new cases of this type of cancer in Brazil.^4^The survival estimates of Brazilian individuals with breast cancer for the period from 2010 to 2014 were five years, so early diagnosis and timely treatment predict greater chances of curing the disease.^1^On the other hand, limited access to diagnostic methods and appropriate and timely treatment, as well as factors related to the knowledge of the disease, result in diagnoses in more advanced stages, worsening the prognosis.^1^The earlier a tumor is identified, the greater the chances of cure.

Research conducted before the pandemic indicates that most patients diagnosed with cancer have a delay of three to six months between the confirmed diagnosis and the start of treatment. The delay between diagnosis and initiation of treatment or care after the onset of symptoms is associated with a worsening of the prognosis of breast cancer, as well as with the repercussion of patient survival.^5^Delays in the diagnosis and treatment of cancer are related to several reasons, whether associated with the access and organization of health services, professionals or patients themselves.^2^The COVID-19 pandemic declared in 2020 possibly also affected the screening, diagnosis and treatment of cancers in the Brazilian population.

Diagnosing and treating breast cancer in a timely manner has always been the public health challenge in Brazil. In the state of Pará, a Brazilian state located in the northern region of the country, territorial aspects such as the distance from specialized health services and the socioeconomic condition of the population are conditioning factors for access to screening, diagnosis and treatment services for various diseases. The COVID-19 pandemic situation has become another barrier to the displacement of women in need of breast cancer screening and monitoring, given the lockdown period and other social isolation measures established by local authorities. After the pandemic was declared by the World Health Organization (WHO), several changes occurred in society, with Brazilian states needing to adopt restrictive measures in order to contain the contagion of the disease. Brazil was one of the four countries with the highest number of confirmed COVID-19 infections, with high rates of transmissibility causing collapse in health services.^5^^,^^6^

A study carried out at a breast imaging center in the state of São Paulo, located in the most developed region of Brazil, identified a 78.9% reduction in imaging tests and breast procedures in the first 90 days of social isolation in 2020, compared to the previous year. The number of breast cancers identified by mammography was 3 times higher than in 2019. Other countries also reported a reduction in breast cancer diagnoses in the first period of the pandemic.^7^A study carried out in Brazil to identify the repercussions of COVID-19 on cancer screening, diagnosis and treatment found a reduction of 42.6% in mammograms, 35.3% in biopsies, 15.7% in oncological surgeries and 0.7% in radiotherapy procedures in 2020 compared to 2019.^8^In the population-based modeling study, the estimated increase in breast cancer deaths due to delayed diagnosis of the disease resulting from the pandemic, within 5 years of detection of the disease was 7.9% to 9.6%.^9^

As the pandemic progressed and the population became more contagious, measures such as isolation, quarantine and social distancing were necessary to control the spread of the disease. Considering that COVID-19 affects individuals with comorbidities, especially the elderly; individuals undergoing cancer treatment constitute a risk group given their decreased immune response as a result of treatment, and protective measures should be reinforced for this public.^10^That access to health services is an obstacle to the treatment of several diseases is nothing new to those who depend on the Brazilian health system or work in it. The poor distribution of health services in the vast territorial extension of the state of Pará reflects inequities in access to services. During the critical period of the pandemic, the difficulty of access to health services by the population was notorious, when the treatment of chronic diseases or elective surgeries was suspended due to the care of patients with COVID-19. There was no physical structure, human resources and equipment to meet the need for care. Based on the observation of the fragility of health services to maintain the monitoring of chronic diseases, including breast cancer, the study aimed to identify whether there was an impact of the COVID-19 pandemic on the screening, diagnosis and treatment of breast cancer in women from 50 years of age in the state of Pará.

The flow of care for any disease must be strategically organized to meet the needs of users, passing through all levels of care. The knowledge and monitoring of care actions provided to the population in periods of crisis and the way this care is being offered, guides nurses in their practices of implementing health policies aimed at the population and in the strategic planning of better actions to be carried out in times of future health crisis. Considering that nurses are the ones who assume the important role of coordinating primary care health programs and also in secondary and tertiary care services, they can benefit from data such as this research, to execute intervention projects aiming at positive impacts on the health of the population.

## Methods

This is a cross-sectional study, with a quantitative approach, with analysis of retrospective data, carried out in DATASUS, with samples of records from 144 municipalities in the Brazilian state. The state of Pará is the second largest territory in Brazil, with an area of 1,247,954.666 km^2^ and an estimated population of 8,513,497 inhabitants, distributed in its 144 municipalities. The state is cut by 20,000 kilometers of rivers, with water and road transport being predominant.

The sample consisted of 102,903 screening and diagnostic tests of women with breast cancer in the state of Pará, aged from 50 years, in addition to the registration of 1,040 treatments. The records were obtained through information systems of the Brazilian national cancer control program (Cancer Information System - SISCAN) and the oncology panel available in the Tabnet 3.2 application. Records of screening tests, diagnoses and treatments of women with breast cancer performed and initiated in the period from January to December 2018, 2019, 2020 and 2021 were selected. In order to verify the volume of exams, screening exams of any periodicity were considered. 

Data collection took place from August 2022 to March 2023 due to data update of the DATASUS system. The following qualitative variables were selected: sex, time of exams and start of treatment. The quantitative variables chosen were: number of screening and diagnostic mammograms, number of cytologies (Fine Needle Aspiration Puncture - FNA), and number of histologies. The data were first organized in spreadsheets in the Microsoft Excel® software, with the pre-pandemic and pandemic periods being related to one of the selected variables (12 months of the year, time interval for the examination and time interval for treatment initiation).

The years 2018-2019 were characterized as a pre-pandemic period, while the years 2020-2021 were characterized as a pandemic period. The time of diagnostic mammography examination and screening were grouped into 2 groups, the first of up to 30 days and the second greater than 30 days; the time of diagnostic examination (FNA, histological) and time of treatment initiation in 3 groups with an interval of 30 days (Up to 30 days, 31 - 60 days, > 60 days). The time interval of the mammography exam comprises the time from the request for the exam to the performance of it, while the time interval of the histology and cytology exam apprehends the time in days from the day of collection to the release of the report by the laboratory, while the treatment start time comprises the interval between the diagnosis signed by the physician and the first therapy instituted. From the organization of the spreadsheets, the absolute frequencies and the percentage variation of exams performed over the months in the periods under study were calculated. Pearson's chi-square test and Williams' G-test were applied to analyze the pre-pandemic and pandemic periods in relation to the variable time to perform the exams and time to start breast cancer treatment in the Brazilian state. The statistical tests were performed in the Bioestat Software, version 5.3, with a significance level of 5%. 

## Results

The total number of breast cancer screening and diagnosis procedures performed in the state of Pará in the context of the pandemic (2020-2021) was 52,284, of which 387 were diagnostic mammograms, 50,914 screening mammograms, 141 cytologies and 842 histologies (Table 1), and 617 records of treatments performed were also observed. 

All procedures performed, whether screening or diagnostic, varied over the periods studied. Diagnostic mammography fell during the pandemic period, while the other exams expressed an increase in their quantities. There was a difference of 244 diagnostic mammograms, 1805 screening mammograms in the pre-pandemic period in relation to the pandemic context. There was a total negative percentage variation of -38.7% in diagnostic mammograms and a positive percentage variation of 3.7% in screening mammograms (table-1). In the monthly relationship between the pre-pandemic and pandemic periods of April and May, there was a lower record of mammogram procedures performed, and this study showed a sharp drop of -33.5%, -37.1% and -88.6%, -90.7% of screening and diagnostic mammograms, respectively, in the months reported above (Table 1). 

**Table 1:** Monthly and annual percentage variation of screening and diagnostic mammography in the state of Pará by period

Month	Screening Mammography			Diagnostic Mammography		
	Pre-pandemic	Pandemic	PV	Pre-pandemic	Pandemic	PV
	*n*	*n*	%	*n*	*n*	%
January	4376	4665	6.6	59	23	-61.0
February	4106	4490	9.4	51	29	-43.1
March	3769	4681	24.2	55	20	-63.6
April	3447	2291	-33.5	44	5	-88.6
May	3444	2167	-37.1	43	4	-90.7
June	3356	2886	-14.0	63	45	-28.6
July	3535	3642	3.0	49	32	-34.7
August	3972	4054	2.1	59	34	-42.4
September	3703	4358	17.7	63	56	-11.1
October	5002	5841	16.8	53	53	0.0
November	5353	6159	15.1	41	33	-19.5
December	5046	5680	12.6	51	53	3.9
Total	49109	50914	3.7	631	387	-38.7

Source: SISCAN. PV: Percentage variation; Pre-pandemic: 2018-2019; Pandemic: 2020-2021; *n*: Absolute number of exams.

Cytological and histological examinations showed a difference of 27 FNA and 77 histologies performed in the pre-pandemic period in relation to the pandemic context, with an even greater drop in May -73.3% and -46.5%, with an increase in October for FNA 220% and in January for histology 69.6% (Table 2). 

**Table 2:** Monthly and annual percentage change in FNA and histology, in the state of Pará, by period

Month	PNA			Histology		
	Pre-pandemic	Pandemic	PV	Pre-pandemic	Pandemic	PV
	*n*	*n*	%	*n*	*n*	%
January	7	20	185.7	56	95	69.6
February	15	8	-46.7	112	73	-34.8
March	6	11	83.3	65	81	24.6
April	7	12	71.4	41	55	34.1
May	15	4	-73.3	86	46	-46.5
June	7	16	128.6	62	69	11.3
July	12	4	-66.7	65	62	-4.6
August	11	16	45.5	38	57	50.0
September	9	12	33.3	76	88	15.8
October	5	16	220.0	52	71	36.5
November	13	10	-23.1	70	92	31.4
December	7	12	71.4	42	53	26.2
Total	114	141	23.7	765	842	10.1

Source: SISCAN. PV: Percentage variation; Pre-pandemic: 2018-2019; Pandemic: 2020-2021; *n*: Absolute number of exams.

In the time interval for exams and treatment initiation, there was a significant difference between the pre-pandemic and pandemic periods, with p values <0.05. Of the total of 1108 registered diagnostic mammography exams, 62% were performed in the pre-pandemic period, 65.5% in the time interval greater than 30 days. While of 100,023 screening mammograms, 50.9% were performed in the pandemic period, with the highest number performed within 30 days. The difference between the periods under analysis was significant, with a *p* value <0.0001 for both types of mammography, based on the application of Pearson's Chi-square test. Of the FNA and histology exams (1862) 55.3% and 52.4%, respectively, were performed in the pandemic period with the highest proportion of exams recorded in the time interval of up to 30 days. For both exams, a statistically significant difference was observed in the relationship between the periods, with p values found, respectively, *p*=0.0286 and *p*<0.0001. To identify whether there was significance in the examination time interval between periods, Williams' G test was applied for FNA and Pearson's Chi-square test for histology (Table 3).

**Table 3:** Time interval for exams, by period, in the state of Pará

Exams	Pre-pandemic			Pandemic			***p*-value**
	< 30 days	31-60 days	>60 days	< 30 days	31-60 days	>60 days	
Diagnostic Mammography	218	413	0	265	122	0	<0.0001*
Screening mammography	33287	5822	0	37546	13368	0	<0.0001*
Total	33505	16235	0	37811	13490	0	
PNA	97	8	9	132	7	2	0286
Histology	504	195	66	622	191	29	< 0.0001
Total	601	203	75	754	198	31	

Source: SISCAN. Pre-pandemic: 2018-2019; Pandemic: 2020-2021; *: Pearson's chi-square test; **: Williams' G test.

As for breast cancer therapy in Pará, 1040 treatments were recorded, of which 59.3% were performed in the pandemic period. The time interval for treatment initiation was greater than 60 days, with a percentage of 66.13% of treatments initiated in this period. There was a statistically significant difference between the periods studied, with a value of p<0.0001. (Table 4).

**Table 4:** Time to start breast cancer treatment, by period, in the state of Pará

Time	**Pre-pandemic (*n*)**	**Pandemic (*n*)**	PV
Up to 30 days	82	67	-18.3
31 - 60 days	124	142	+14.5
By 60 Days	217	408	+88
Total	423	617	+45.9

Source: SISCAN. Pre-pandemic: 2018-2019; Pandemic: 2020-2021

## Discussion

The COVID 19 pandemic changed the global scenario in the economic, social and health context. The high number of people infected and the rapid spread of the virus had repercussions on the structuring, organization and delivery of health services around the world. Screening and monitoring procedures for several chronic non-communicable diseases, previously already weakened at national level, suffered harsh consequences, leaving patients unassisted. Studies carried out in Brazil and other countries indicate a negative impact of the pandemic on the screening, diagnosis and treatment of several cancers, including breast cancer. Analyses of the effect of the pandemic on breast cancer screening in Brazil, through the registration of mammograms, observed a reduction in the total number of this procedure performed in the pandemic period.^12^

In the United States, at the peak of the pandemic, a reduction of -85% in breast cancer examinations was identified.^13^While in an American network with a registry of 28 million patients, a reduction of -89.2% in breast cancer screening was also observed.^14^A study conducted in the regions of Brazil showed a decrease of -44% in the total number of screening mammograms performed in the country and -25% in the northern region.^15^While another survey showed a reduction of -40% in the total number of mammograms in 2020, screening mammograms were the most affected with a reduction of up to -41.65% and diagnostics -21.84%.^12^This investigation also states that although all Brazilian states showed a drop in the number of exams, Pará was one of the states with the lowest reduction in the volume of exams performed, - 2.01%.^12^

A Danish survey observed a drop in breast cancer screening shortly after the pandemic was decreed, even though the country maintained the breast cancer screening procedure in the pandemic period.^16^While in an Irish study 30.5% of women reported experiencing disruption in breast cancer services, and thus impacts of COVID-19.^17^In the state of Pará, through this study, a reduction in the registration of screening and diagnostic tests performed in 2020 was identified. This drop in the proportion of tests coincided with the first year of the pandemic in Brazil, when in March, the WHO declared the COVID-19 pandemic. The highest incidence of cases and deaths from COVID-19 recorded in the state of Pará occurred in April and May, months evidenced in the present study, in addition to June, as those with the highest drop in procedures performed compared to the previous year. In 2021, there was resumption in the performance of the exams; therefore, there was an increase in the number of exams performed. In the relationship between the pre-pandemic and pandemic periods, there was a greater registration of screening, FNA and histological mammography, and on the other hand, a decrease in diagnostic mammography performed in the period considered as pandemic. There was a difference of 1805 screening mammograms, 27 FNA, 77 histological and 244 diagnostic mammograms. 

Although studies indicate that breast cancer actions in Brazil and around the world have been affected by the COVID-19 pandemic, the current study points out that in the state of Pará there was an increase in the number of exams carried out during the period considered pandemic. This divergence may be related to the relaxation of restrictive measures in the state, an attempt to normalize health services and the resumption of procedures carried out in 2021, which with an increase in the production of such exams had an impact on the gross number of exams carried out in the pre-pandemic and pandemic periods, the latter with a greater number of exams carried out. Given the large number of people infected, the WHO's main recommendation was social isolation in order to reduce the spread of the disease and mitigate the impact of the pandemic on the health system. Due to the concern of exposing patients to the COVID virus and the development of severe forms of the disease in cancer patients, the National Cancer Institute (INCA) recommended that health professionals postpone screening for breast cancer and evaluate each case for the real need to perform it.^18^In line with the recommendations of the WHO, the state of Pará decreed red flagging, adopting as measures to curb the advance of COVID; the restrictive time for the movement of people on the streets, restriction of the number of people in certain establishments, social distancing, among others. The overload on health services required the prioritization of care for COVID patients to the detriment of patients with chronic non-communicable diseases, including cancer. Health services were instructed to postpone consultations and examinations for a time of reduction of restrictions, having to evaluate the risks and benefits of elective procedures. In March 2021, the restrictive measures were again implemented due to the increase in new cases.

In the midst of the health crisis caused by the pandemic, the substantial role of nurses in the identification and active search for patients at high risk for breast cancer stands out, since screening is contraindicated by INCA. On March 26, 2020, the Federal Council of Nursing (COFEN) authorized and standardized the nursing tele-consultation for clarification, referrals and guidance to patients, facilitating the care process. Breast cancer patients need an individualized evaluation, access to information and safe assistance. Thus, through tele-consultation, nurses had the opportunity to ensure the safe access of cancer patients to health services, free of risks, either through hygiene guidelines, use of masks and reorganization of the care environment.

Regarding the time interval for performing screening tests and diagnoses of breast cancer, in the state of Pará, the tests were performed in their largest proportion in the time interval of up to 30 days, in both the pre-pandemic and pandemic periods. In order to ensure timely diagnosis and timely treatment, since 2019 Law number 13.896, directs that tests to confirm malignant neoplasm should be performed within a maximum of 30 days.^(19, 20)^ Early diagnosis and therefore timely treatment provides for greater chances of curing breast cancer. An English survey estimated an increase of 2.1 to 9.6% in the number of deaths in the medium (1 year) and long term (5 years) from breast cancer due to delayed diagnosis in the pandemic period.^9^The reduced number of diagnostic tests performed in the state of Pará implies that women seek health services when the prognosis is no longer positive, which can be seen in the long term. The delay in diagnosis is related to the health system itself regarding its organization for the population's access to services and its own diagnostic capacity. Community awareness of the identification of signs and symptoms of the disease and timely service-seeking behavior is another influential factor in early diagnosis.

In the present study, the state had a time greater than 60 days to start breast cancer treatment, in addition to a greater record of cases treated in the pandemic period. Another study, which used data from the oncology panel, showed untreated patients within the 60-day period.^21^The treatment of cancers is guaranteed free of charge by the Brazilian health system, and must be started within 60 days from the day of diagnosis confirmed by a report, whatever the therapeutic need.^19^Delays longer than 60 days in breast cancer therapy result in worsening patient survival.^22^

A Brazilian retrospective cohort study conducted between 2000 and 2011 showed that the time interval between diagnosis and treatment of breast cancer in the North region was 49 days. The same study also reveals that the North region has shown the worst results in relation to mammography, biopsies, early diagnosis and access to treatment.^22^In the state of Rio de Janeiro, located in the southeastern region of Brazil , the mean time to start breast cancer treatment was 206 days.^23^^)^

Brazilian regional differences were highlighted regarding the structure of oncological services and mammographic coverage, also suggesting that the delay in the initiation of breast cancer treatment may be justified by the higher incidence of breast cancer in recent years and consequently greater demand for treatments not accompanied by the organization of oncology services.^22^ Geographic disparities and the distribution of health services in the extensive territory of Pará, reinforce the aforementioned observations. The estimated incidence of breast cancer for the three-year period 2023-2025 is 24.99 new cases per 100,000 women in the North Region and 22.83% for 2023 in the state of Pará.^4^

Due to the large territorial extension of the state of Pará, the inequality in the distribution of health services and access of the population, in addition to socioeconomic and cultural issues, reflect in the performance of tests, time of diagnosis and treatment of women with cancer. The distance traveled by women with breast cancer to obtain access to health services confirms the fragility of the oncological care network in the state, exacerbated in the midst of the health crisis. The pandemic has brought to light several weaknesses in the health system of the countries. The response time, the rapid speed of transmission of the virus and the forms of treatment of the disease have become a challenge to Brazilian and Pará public health.

This study shows us, statistically, that there was no interference of the COVID-19 pandemic in the actions of breast cancer in the state; on the other hand, it is observed that in this period there was no orientation of reorganization of oncological services by the Ministry of Health (MH) and INCA so that the actions of screening and diagnosis were maintained, being the responsibility of the state and municipalities to develop strategies according to the demands of their oncology services. It is necessary to carry out studies that point out the distribution, coverage and organization of oncological services in Brazil, in order to reflect the promotion of strategies that minimize the disparities in the population's access to health services so that all the principles of the Brazilian health system are in fact met. For if in a future health crisis we neglect once again a disease that is the first cause of death in the world, we will condemn the population to a silent death. This study was limited by the fact that the data source is constantly updated and provides divergent data in terms of quantity. The oncology panel was only made available to managers in May 2019, and manual verification of the data obtained was required.

Conclusion From the data obtained, it was possible to achieve the objectives proposed for the study. The results indicated an important increase in the number of screening and diagnostic procedures for breast cancer in the pandemic period, with the exception of diagnostic mammography. However, when considering the values of (p), it appears that the COVID-19 pandemic did not interfere with breast cancer health actions in the state of Pará-Brazil.
